# Safety of COVID-19 Vaccination in Patients With Breast Cancer: Cross-Sectional Study in China

**DOI:** 10.2196/46009

**Published:** 2023-12-07

**Authors:** Shaohua Zhang, Jianbin Li, Ruonan Xu, Qianjun Chen, Gang Sun, Ying Lin, Yali Cao, Yiding Chen, Cuizhi Geng, Yuee Teng, Jianyun Nie, Xinzheng Li, Guiying Xu, Xinlan Liu, Feng Jin, Zhimin Fan, Ting Luo, Hong Liu, Fu-sheng Wang, Zefei Jiang

**Affiliations:** 1 Fifth Medical Center of Chinese People's Liberation Army General Hospital Beijing China; 2 Beijing Institute of Biotechnology Academy of Military Medical Sciences Beijing China; 3 Guangdong Provincial Hospital of Traditional Chinese Medicine Guangdong China; 4 Cancer Hospital Affiliated to Xinjiang Medical University Xinjiang China; 5 The First Affiliated Hospital of Sun Yat-sen University Guangdong China; 6 Nanchang Third Hospital Jiangxi China; 7 The Second Affiliated Hospital of Medical College of Zhejiang University Zhejiang China; 8 Fourth Hospital of Hebei Medical University Hebei China; 9 The First Hospital of China Medical University Liaoning China; 10 Yunnan Cancer Hospital Yunnan China; 11 Shanxi Cancer Hospital Shanxi China; 12 Jilin Cancer Hospital Jilin China; 13 General Hospital of Ningxia Medical University Ningxia China; 14 The First Hospital of Jilin University Jilin China; 15 Sichuan Uniersity Huaxi Campus Sichuan China; 16 Tumor Hospital of Tianjin Medical University Tianjin China

**Keywords:** breast cancer, COVID-19 vaccines, patients reported adverse events, healthy population, vaccine safety

## Abstract

**Background:**

The widespread use of vaccines against the novel coronavirus disease (COVID-19) has become one of the most effective means to establish a population immune barrier. Patients with cancer are vulnerable to COVID-19 infection, adverse events, and high mortality, and should be the focus of epidemic prevention and treatment. However, real-world data on the safety of vaccines for patients with breast cancer are still scarce.

**Objective:**

This study aims to compare the safety of COVID-19 vaccines between patients vaccinated before or after being diagnosed with breast cancer.

**Methods:**

Patients with breast cancer who sought medical advice from October 2021 to December 2021 were screened. Those who received COVID-19 vaccines were enrolled in this study to analyze the safety of the vaccines. The primary outcome was patient-reported adverse events (AEs). All events after vaccine injection were retrospectively documented from the patients.

**Results:**

A total of 15,455 patients with breast cancer from 41 hospitals in 20 provinces in China were screened, and 5766 patients who received COVID-19 vaccines were enrolled. Of those enrolled, 45.1% (n=2599) of patients received vaccines before breast cancer diagnosis, 41.3% (n=2379) were vaccinated after diagnosis, and 13.6% (n=784) did not known the accurate date of vaccination or cancer diagnosis. Among the patients vaccinated after diagnosis, 85.4% (n=2032) were vaccinated 1 year after cancer diagnosis and 95.4% (n=2270) were vaccinated during early-stage cancer. Of all 5766 vaccinated patients, 93.9% (n=5415) received an inactivated vaccine, 3.7% (n=213) received a recombinant subunit vaccine, and 2.4% (n=138) received other vaccines, including adenovirus and mRNA vaccines. In the first injection of vaccines, 24.4% (n=10, 95% CI 11.2-37.5) of patients who received an adenovirus vaccine reported AEs, compared to only 12.5% (n=677, 95% CI 11.6-13.4) of those who received an inactivated vaccine. Patients with metastatic breast cancer reported the highest incidence of AEs (n=18, 16.5%, 95% CI 9.5-23.5). Following the second injection, patients who received an inactivated vaccine (n=464, 8.7%, 95% CI 8.0-9.5) and those who received a recombinant vaccine (n=25, 8.7%, 95% CI 5.5-12.0) reported the same incidence of AEs. No significant differences in patient-reported AEs were found between the healthy population and patients with breast cancer (16.4% vs 16.9%, respectively); the most common AEs were local pain (11.1% vs 9.1%, respectively), fatigue (5.5% vs 6.3%, respectively), and muscle soreness (2.3% vs 3.6%, respectively). The type of vaccine and time window of vaccination had little impact on patient-reported AEs.

**Conclusions:**

Compared with patients vaccinated before breast cancer diagnosis, there were no significant differences in patient-reported AEs in the patients vaccinated after diagnosis. Thus, it is safe for patients with breast cancer, especially for those in the early stage, to receive COVID-19 vaccines.

**Trial Registration:**

Chinese Clinical Trial Registry ChiCTR2200055509; https://tinyurl.com/33zzj882

## Introduction

The outbreak of COVID-19 has had a huge impact on the lives and work of people worldwide [1-3]. Although several drugs have been authorized for emergency use by many countries [4,5], inaccessibility and strong demand have limited their widespread use during the pandemic. The establishment of a population immune barrier through mass vaccination with COVID-19 vaccines may be the one of the most effective means to end the pandemic at this moment [6]. However, for certain populations, safety concerns of vaccines may have negative impacts on widespread vaccination, thus weakening the process of herd immunity. In this process, vaccination of specific patients is an important part of epidemic prevention and control [7,8].

Cancers are the second leading cause of death worldwide, with high morbidity and mortality rates in the general population. In the setting of the SARS-CoV-2 pandemic, patients with cancer are at high risk of infection, and the safety of these patients after COVID-19 vaccination is an important consideration. Aberrant immune responses in the context of underlying cancer and the use of anticancer therapies may contribute to impaired immune responses and altered reactogenicity following immunization against SARS-CoV-2 [9,10]. Therefore, patients with tumors have had a high risk of infection, high incidence of serious events, and high mortality in the COVID-19 pandemic [11]. As a special group, there is still confusion on whether the safety of COVID-19 vaccines in such patients is the same as in healthy individuals because of their immunosuppression [12,13].

For patients with breast cancer, there has been a history of receiving vaccines for cancer treatment [14]. However, the COVID-19 vaccination rate remains low in China due to inadequate health education, the lack of vaccine safety data, and even some other social status influences rather than vaccine shortages [15-17]. The fear of delaying cancer therapy has surpassed the fear of COVID-19 infection [18]. To better understand why some patients with cancer are unwilling to receive vaccines and the safety profiles of those who have received vaccines, we conducted this real-world study, called CSCO BC NCP-02, from cancer centers across China [19]. Notably, we found that half of the patients in the study received COVID-19 vaccines before cancer diagnosis. This allowed us to compare the differences in adverse events (AEs) between patients who were vaccinated before or after breast cancer diagnosis, thus providing more data to encourage patients with breast cancer to be vaccinated with a COVID-19 vaccine. We aimed to describe the clinical features and AEs of patients with breast cancer and identify risk factors associated with AEs in these patients.

## Methods

### Sample and Data

This was a cross-sectional study initiated by the Chinese Society of Clinical Oncology breast cancer (CSCO BC) committee. The inclusion criteria were as follows: (1) patients who received at least 1 dose of a COVID-19 vaccine, (2) patients diagnosed as having invasive breast cancer, (3) inpatients who visited the participating hospital from November 2021 to December 2021, and (4) patients with more than 3 months of overall survival. The exclusion criteria were as follows: (1) patients with microinvasion of ductal carcinoma in situ breast cancer and (2) patients with a second primary tumors.

Different types of vaccines were recorded. Dosing regimens were as follows: 1 dose for adenovirus vectored vaccines, 2 doses for inactivated viral vaccines and mRNA vaccines, and 3 doses for recombinant vaccines.

### Procedures

Inpatients with breast cancer who were vaccinated were asked to provide retrospective answers through self-reported questionnaires. For highly professional medical issues, physicians were asked to assist the patients to complete questionnaires. Differences for those who received vaccines before cancer diagnosis versus after cancer diagnosis were compared according to the time of cancer diagnosis and vaccination. Patients were recruited in hospitals by the CSCO BC committee. By adopting a competitive enrollment method, only hospitals that contributed more than 20 valid questionnaires by the cutoff date were included.

Through telephone follow-ups or questionnaire surveys, data including epidemiology, demography, clinical information, previous and current therapies, type of vaccines, and patient-reported AEs and their severity after vaccination were collected nationwide. Medical workers of participating hospitals were responsible for data collection, and at least 1 professional doctor from each hospital was responsible for data identification. To ensure the quality of the data, we reviewed all the data, and we retrieved missing data by answering questions. All the data were checked by 2 physicians.

### Ethical Considerations

This study was registered (ChiCTR2200055509) and was approved by the ethics board of the Fifth Medical Center of the Chinese People's Liberation Army General Hospital (KY-2022-7-45-1). Oral informed consent was obtained from participants, and secondary analysis was allowed without additional consent. The study data were deidentified. No compensation was involved in this study.

### Measures of Variables

The outcome was the safety of the COVID-19 vaccines in patients with breast cancer, which was mainly calculated by dividing the number of AEs by the total population. Another observed outcome was the incidence of AEs, which was calculated by dividing the number of patients with AEs by the total population. Patients were divided into 3 groups according to their time of vaccination and cancer diagnosis: vaccinated before cancer diagnosis, vaccinated after diagnosis, or unknown. AEs were analyzed according to the vaccination time and different doses of the vaccines.

### Data Analysis Procedure

SPSS 21.0 (IBM) was used for data statistics. Pearson χ² was used for data analysis and a 2-way ANOVA was used to detect data differences. Frequency tables were analyzed using the χ² test or Fisher exact test. A multivariable logistic regression model was fitted to examine possible differences in AEs. Odds ratios (ORs) and 95% CIs were calculated with Mantel-Haenszel models. A 2-sided α<.05 was considered statistically significant. GraphPad Prism v6.0 was used as the drawing software.

## Results

**Figure 1:**
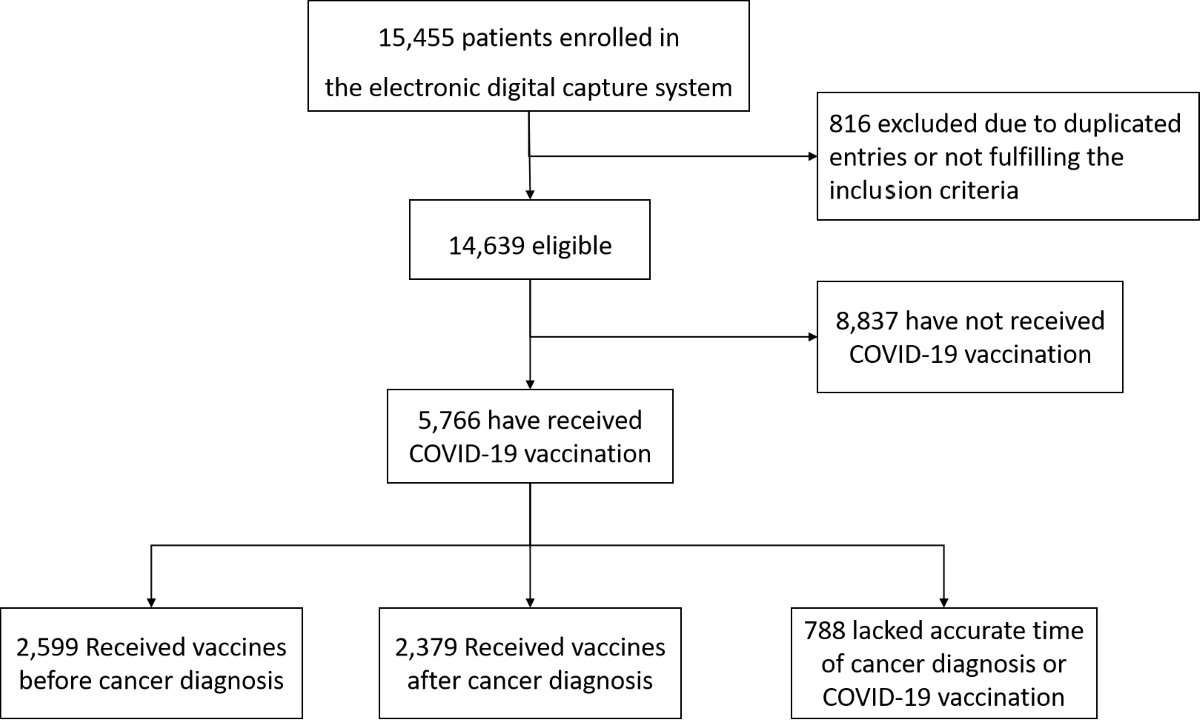
Flow chart of the study.

A total of 15,455 patients from 41 hospitals in China were screened (Figure 1), and 5766 patients were eligible in this analysis, of which 45.1% (n=2599) were vaccinated before cancer diagnosis, 41. 2% (n=2379) were vaccinated after diagnosis, and 13.7% (n=788) lacked an accurate diagnosis time or vaccination time. The baseline characteristics of the patients are detailed in Table 1.

**Figure 2:**
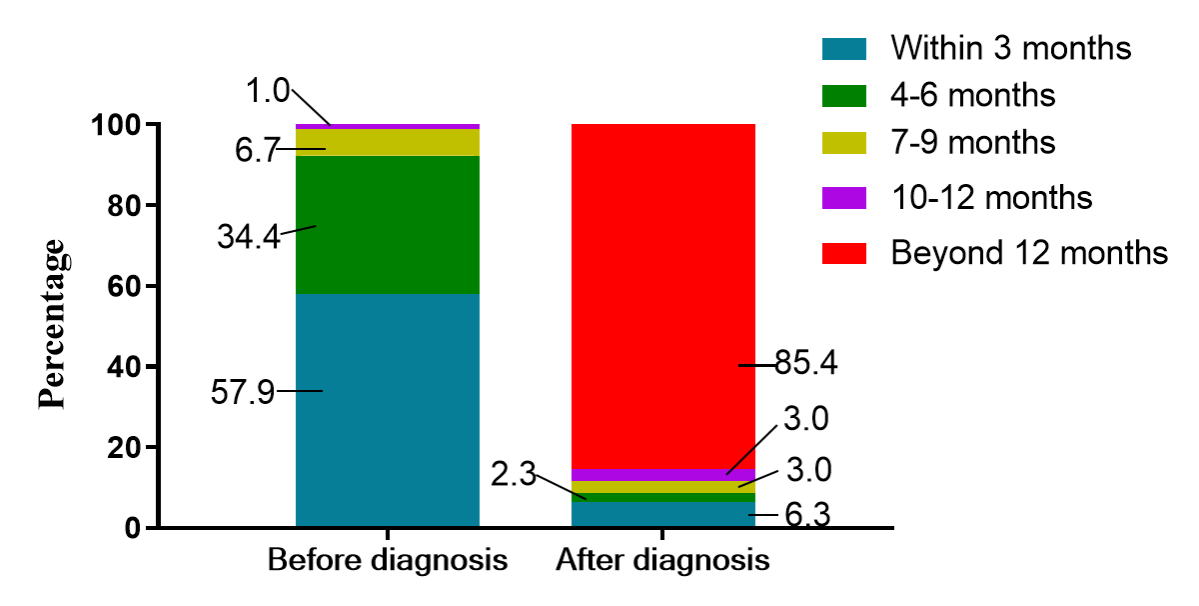
Time interval between COVID-19 vaccination and cancer diagnosis. Those without accurate dates of breast cancer diagnosis or COVID-19 vaccination were excluded.

Among the patients vaccinated before breast cancer diagnosis, 57.9% (n=1505) were diagnosed with breast cancer 1-3 months after vaccination and 34.4% (n=963) were diagnosed 4-6 months after vaccination. Among the patients vaccinated after breast cancer diagnosis, 85.4% (n=2032) received the vaccine 1 year after cancer diagnosis (Figure 2).

**Figure 3:**
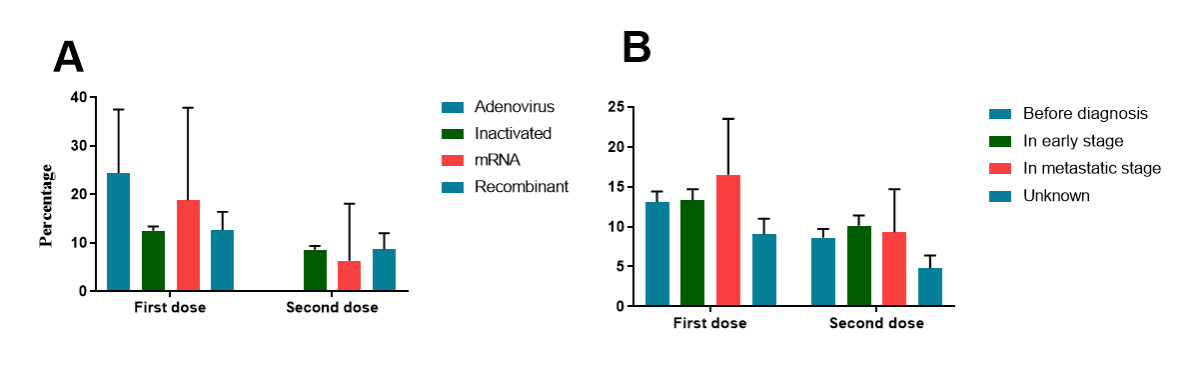
Distribution of adverse events. (A) Incidence of adverse events in patients vaccinated with different vaccines. (B) Incidence of adverse events in patients vaccinated at different stages of breast cancer.

Of all the patients, 93.9% (n=5415) received an inactivated vaccine, 3.7% (n=213) received a recombinant vaccine, and 2.4% (n=138) received other vaccines, including adenovirus and mRNA vaccines. Statistical analysis showed that more patient-reported AEs occurred following the first dose than following the second dose (Figure 3A). Following the first injection, 24.4% (n=10, 95% CI 11.2-37.5) of patients who received an adenovirus vaccine reported AEs compared to 12.5% (n=677, 95% CI 11.6-13.4) for those who received an inactivated vaccine. Following the second dose, patients who received an inactivated vaccine (n=464, 8.7%, 95% CI 8.0-9.5) or a recombinant vaccine (n=25, 8.7%, 95% CI 5.5-12.0) reported the same incidence of AEs (Figure S1 in Multimedia Appendix 1).

Considering the timing of vaccination, of the patients vaccinated before diagnosis, 13.1% (n=340, 95% CI 11.8-14.4) and 8.6% (n=219, 95% CI 7.5-9.7) reported AEs following their first and second injections, respectively (Figure 3B). The cohort included patients in the early stage and patients with metastatic breast cancer. In the early stage, 10.1% (n=227, 95% CI 8.9-11.4) of patients reported AEs following the second dose. Of the patients with metastatic breast cancer, 16.5% (n=18, 95% CI 9.5-23.5) reported AEs after the first dose and 9.3% (n=10, 95% CI 3.8-14.7) after the second dose.

Of all the patients included in this study, a total of 16.4% (n=427) and 16.9% (n=402) of patients vaccinated before or after cancer diagnosis, respectively, reported AEs (Table 2). The most common AEs were local pain (11.1% vs 9.1%, respectively), fatigue (5.5% vs 6.3%, respectively), and muscle soreness (2.3% vs 3.6%, respectively). Other reported AEs included local swelling, local induration, headache, fever, joint pain, and nausea, among others. The incidence of grade 3/4 AEs was relatively low. The highest incidence was for fever, which was reported by 0.4% (n=11) of patients vaccinated before diagnosis and 0.2% (n=5) of those vaccinated after diagnosis.

To identify the factors associated with AEs, a multivariate analysis was conducted (Table S1 in Multimedia Appendix 1). The type of vaccine (odds ratio [OR]=0.937, 95% CI 0.689-1.273) and the time window of vaccination (OR=0.919, 95% CI 0.785-1.077) had little impact on AEs. Patients with low educational degrees reported a lower incidence of AEs (OR=0.794, 95% CI 0.680-0.927) (Table S2 in Multimedia Appendix 1).

**Table 1:** Baseline characteristics of vaccinated patients.

Characteristic		Vaccinated before diagnosis (n=2599), n (%)	Vaccinated after diagnosis (n=2379), n (%)	Timing unknown (n=788), n (%)	Total (n=5766), n (%)
**Age (years)**					
	≤45	904 (34.8)	935 (39.3)	211 (26.8)	2050 (35.6)
	>45	1632 (62.8)	1345 (56.5)	431 (54.7)	3408 (59.1)
	Unknown	63 (2.4)	99 (4.2)	146 (18.5)	308 (5.2)
**Chronic illness**					
	No	2143 (82.5)	1841 (77.4)	657 (83.4)	1107 (19.2)
	Yes	456 (17.5)	538 (22.6)	131 (16.6)	4659 (80.8)
**Level of education**					
	Without bachelor degree	1575 (60.6)	1543 (64.8)	462 (58.6)	3580 (62.1)
	With bachelor degree	1013 (39)	812 (34.1)	210 (26.6)	2035 (35.3)
	Unknown	11 (0.4)	24 (1)	116 (14.7)	151 (2.5)
**T stage**					
	T1-2	1927 (74.1)	1612 (67.8)	322 (40.8)	3861 (67)
	T3-4	199 (7.7)	112 (4.7)	25 (3.2)	336 (5.8)
	Unknown	473 (18.2)	655 (27.5)	441 (56)	1569 (27.2)
**N stage**					
	N0	1115 (42.9)	1096 (46.1)	215 (27.3)	2426 (42.1)
	N1-3	1051 (40.4)	727 (30.6)	139 (17.6)	1917 (33.2)
	Unknown	433 (16.7)	556 (23.3)	434 (55.1)	1423 (24.6)
**HR^a^**					
	Positive	1523 (58.6)	1755 (73.8)	246 (31.2)	3524 (61.1)
	Negative	626 (24.1)	439 (18.5)	97 (12.3)	1162 (20.2)
	Unknown	450 (17.3)	185 (7.7)	445 (56.5)	1080 (18.7)
**HER2^b^**					
	Positive	825 (31.7)	618 (26)	118 (15)	1561 (27.1)
	Negative	1324 (50.9)	1331 (55.9)	225 (28.5)	2880 (50)
	Unknown	450 (17.3)	430 (18.1)	445 (56.5)	1325 (22.9)
**Neoadjuvant**					
	Yes	709 (27.3)	373 (15.7)	95 (12.1)	1177 (20.4)
	No	1890 (72.7)	2006 (84.3)	693 (87.9)	4589 (79.6)
**Surgery**					
	Yes	2089 (80.4)	2295 (96.5)	403 (51.1)	4787 (83)
	No	510 (19.6)	84 (3.5)	385 (48.9)	979 (17)
**Adjuvant**					
	Yes	1704 (65.6)	2105 (88.5)	284 (36)	4093 (71)
	No	895 (34.4)	274 (11.5)	504 (64)	1673 (29)
**Metastatic**					
	Yes	128 (4.9)	203 (8.5)	11 (1.4)	342 (5.9)
	No	2471 (95.1)	2176 (91.5)	777 (98.6)	5424 (94.1)

^a^HR: hormone receptor.

^b^HER2: human epidermal growth factor receptor 2.

**Table 2:** Safety analysis of COVID-19 vaccines in the enrolled patients.

			Vaccinated before diagnosis (n=2599), n (%)	Vaccinated after diagnosis (n=2379), n (%)	Timing unknown (n=788), n (%)
Adverse events			427 (16.4)	402 (16.9)	106 (13.5)
**Local reactions**					
	**Local pain**				
		Any	288 (11.1)	218 (9.1)	38 (4.8)
		Grade 3/4	2 (0.1)	3 (0.1)	0 (0)
	**Local swelling**				
		Any	43 (1.7)	33 (1.4)	7 (0.9)
		Grade 3/4	2 (0.1)	2 (0.1)	0 (0)
	**Subcutaneous nodules**				
		Any	32 (1.2)	9 (0.4)	6 (0.8)
		Grade 3/4	4 (0.1)	1 (<0.1)	0 (0)
**Systemic reactions**					
	**Fever**				
		Any	29 (1.1)	25 (1)	6 (0.6)
		≥38 °C	11 (0.4)	5 (0.2)	3 (0.3)
	**Headache**				
		Any	48 (1.8)	41 (1.7)	8 (1)
		Grade 3/4	2 (0.1)	1 (<0.1)	0 (0)
	**Fatigue**				
		Any	142 (5.5)	150 (6.3)	36 (4.6)
		Grade 3/4	3 (0.1)	0 (0)	0 (0)
	**Muscle soreness**				
		Any	61 (2.3)	87 (3.6)	13 (1.7)
		Grade 3/4	2 (0.1)	1 (<0.1)	0 (0)
	**Joint pain**				
		Any	18 (0.7)	24 (1)	4 (0.5)
		Grade 3/4	2 (0.1)	3 (0.1)	0 (0)
	**Nausea**				
		Any	27 (1)	22 (0.9)	2 (0.3)
		Grade 3/4	2 (0.1)	1 (<0.1)	0 (0)
	**Loss of appetite**				
		Any	12 (0.5)	10 (0.4)	4 (0.5)
		Grade 3/4	2 (0.1)	1 (<0.1)	0 (0)
	**Anaphylaxis**				
		Any	6 (0.2)	15 (0.6)	0 (0)
		Grade 3/4	0 (0)	0 (0)	0 (0)
	**Dizzy**				
		Any	4 (0.2)	12 (0.5)	0 (0)
		Grade 3/4	0 (0)	0 (0)	0 (0)
	**Disturbance in respiration**				
		Any	0 (0)	5 (0.2)	0 (0)
		Grade 3/4	0 (0)	0 (0)	0 (0)
	**Breast pain**				
		Any	5 (0.2)	1 (<0.1)	0 (0)
		Grade 3/4	0 (0)	0 (0)	0 (0)
	**Thirsty**				
		Any	6 (0.2)	0 (0)	0 (0)
		Grade 3/4	0 (0)	0 (0)	0 (0)
	**Diarrhea**				
		Any	1 (<0.1)	2 (0.1)	0 (0)
		Grade 3/4	0 (0)	1 (<0.1)	0 (0)
	**Angina pectoris**				
		Any	1 (<0.1)	4 (0.2)	0 (0)
		Grade 3/4	0 (0)	1 (<0.1)	0 (0)
	**Others**				
		Any	23 (0.9)	20 (0.8)	0 (0)
		Grade 3/4	0 (0)	0 (0)	0 (0)

## Discussion

### Principal Findings

This was a real-world study to compare patient-reported AEs among patients vaccinated before or after breast cancer diagnosis. The results have several implications. First, most patients vaccinated after breast cancer diagnosis received their vaccines 1 year after diagnosis, indicating that most patients with early breast cancer received vaccines after chemotherapy due to the short therapeutic period. Second, the incidence of AEs in patients with early-stage breast cancer was lower than in those with metastatic breast cancer. When compared with patients vaccinated before diagnosis, the reported AEs from patients vaccinated after diagnosis was not increased. Third, patients reported a higher incidence of AEs following their first dose compared to following their second dose. The most common AEs were local pain and fatigue. The type of vaccine and time window of vaccination had little impact on AEs.

In this study, of those vaccinated before diagnosis, 90% were diagnosed with breast cancer within 6 months of vaccination. Ipsilateral axillary swelling was one of most frequently reported local reactions to the COVID-19 vaccine, occurring in 11.6% and 16% of recipients following the first and second dose, respectively [20,21]. The lymphadenopathy caused by vaccination may encourage them to seek specialist treatment, which was an important reason for the higher rate of positive lymph nodes on diagnosis in those patients [22]. For patients vaccinated after their diagnosis with breast cancer, most of them received a COVID-19 vaccine 1 year after their diagnosis. Due to the fact that 91.5% of these patients were in the early stage, this contributed to a large proportion of patients being vaccinated without any chemotherapy, which echoed the recommendations in the Chinese expert consensus [23] and mitigated the negative impact on the immune system from chemotherapy [24,25]. The promotion of the consensus does have potential benefits for elevating the rate of vaccination for patients with cancer [26].

In terms of different kinds of vaccines, the majority of patients in this study chose inactivated vaccines (eg, Beijing Biotech, Wuhan Biotech, and Sinovac). Compared with other types of vaccines [27,28], inactivated vaccines presented relatively good safety data in patients with breast cancer [29], although no significant result was shown in the multivariate analysis. We also found that there were significant differences in the incidence of AEs in patients with breast cancer at different stages. Compared with patients in the early stage, the incidence of AEs was higher in late-stage patients after the first dose of vaccine. For one thing, the overlap of the toxicity from the vaccine and cancer treatment in advanced breast cancer may increase the incidence of AEs [30,31]. For another, considering the complexity of antigens, the immune response may have been more aggressive, which can also lead to a significant increase in AEs [32].

The common AEs in this study included local pain, fatigue, muscle soreness, local swelling, local induration, headache, and fever. The incidence of AEs in this study was much lower than in a published randomized controlled study [33]. In our multivariable analysis, we found that the time window of vaccination and type of vaccine had little influence on AEs. Notably, patients with different education levels exhibited a significant difference in the incidence of AEs; patients with a low degree of education had a lower incidence of AEs. The limitation of real-world studies attributed to the lack of effective guidance during the investigation and incomplete data from case report forms may be one of the reasons [34]. To avoid declines and delays in cancer treatment after COVID-19 quarantine restrictions [35], patients might conceal some discomfort from their physicians. During cancer therapy, some AEs might be regarded as treatment-related AEs and ignored by patients and clinicians. In addition, patients with severe AEs might be transferred to a specialist hospital rather than a cancer hospital, which can also result in an underestimated incidence of AEs in this study.

As a retrospective real-word study, inevitable selection bias is another limitation. Over 90% of patients received an inactivated vaccine, so the safety data of other vaccines in patients with breast cancer might be premature. We also paid less attention to the efficacy of vaccines, and the originally planned follow-up of infections has also been terminated due to indiscriminative infection of COVID-19 after the end of quarantine restrictions in China since December 2022. Fortunately, we have collected blood samples from some of the patients to explore changes in antibody titers, which might be a remaining effective alternative indicator to evaluate the efficacy of vaccines [36,37]. Additionally, owing to the high possibility of selection bias and measurement bias, although no data support the notion that COVID-19 vaccination would induce breast cancer, much importance should be attached to this kind of population.

### Conclusions

Despite the limitations, our retrospective study highlights the safety of COVID-19 vaccination in patients with breast cancer, and these patients are recommended to receive COVID-19 vaccines, just like healthy individuals. Much attention should be paid to the first injection of vaccines. While limited by retrospective data, more evidence from high quality, double-blind, randomized control trials are expected.

## Appendix

Multimedia Appendix 1 Supplementary figure and tables.

## Abbreviations

AE: adverse events
CSCO BC: Chinese Society of Clinical Oncology breast cancer
OR: odds ratio

## Data Availability

Access to the data set supporting the findings of this study can only legally be accessed on acceptance from the Chinese Society of Clinical Oncology breast cancer database collaborative group. Interested parties can contact the corresponding author for further information. We confirm that others would be able to access these data in the same manner as the authors. We also confirm that the authors do not have any special access privileges that others would not have.
