# Supplementary material for: Safety of COVID-19 Vaccination in Patients With Breast Cancer: Cross-Sectional Study in China
Source: JMIR Public Health Surveill. 2023 Dec 7;9:e46009. doi: 10.2196/46009 (PMC10739232; doi:10.2196/46009)
Supplement: Multimedia Appendix 1 [file publichealth_v9i1e46009_app1.docx]

Figure S1. AEs for different types of vaccines


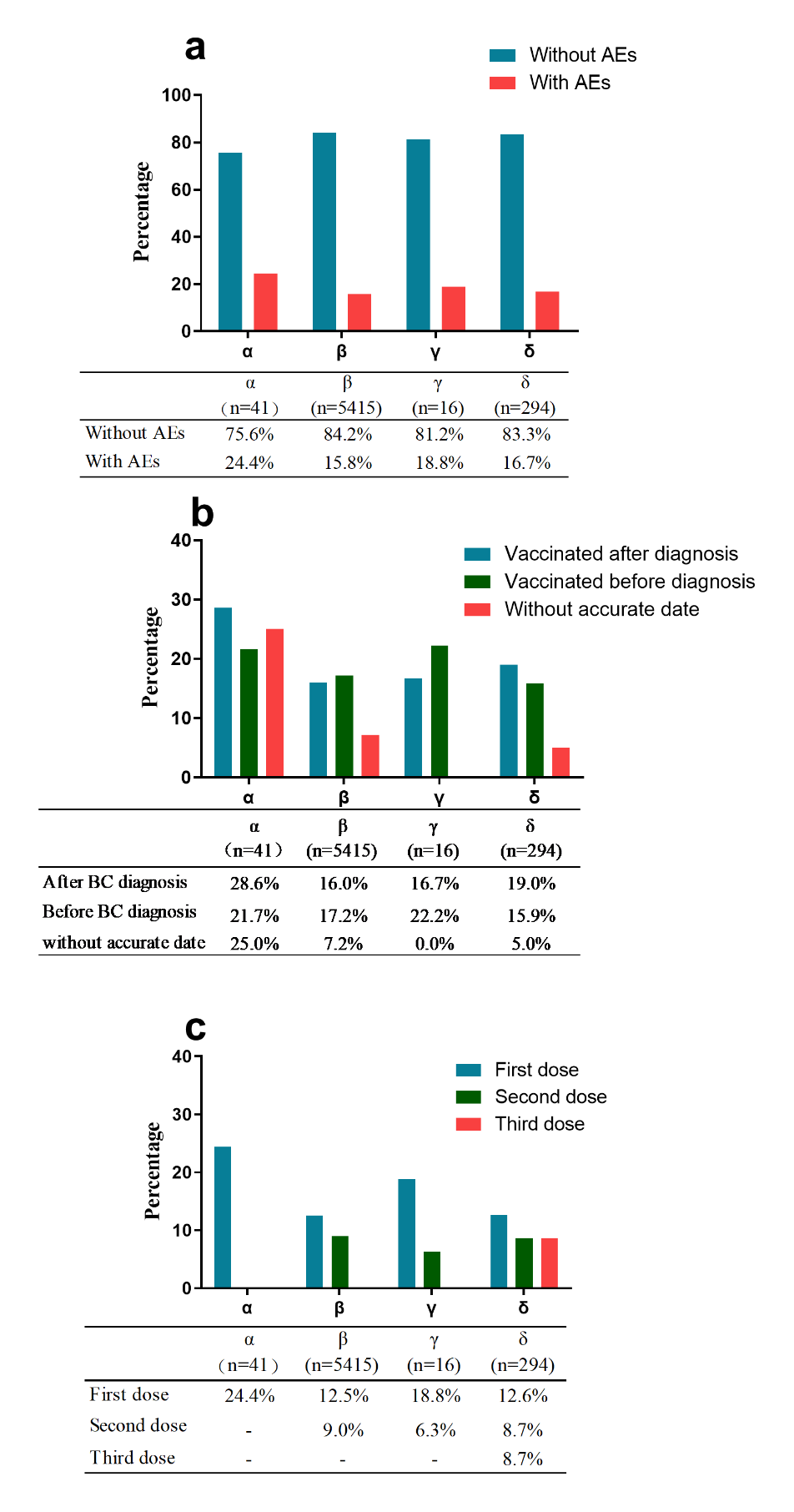


α: adenovirus vaccine. β, inactivated vaccine. γ,mRNA vaccines. δ, recombinant vaccine

Table S1 Safety analysis of inactivated COVID-19 vaccine in enrolled patients.

|  | First dose  N=5415 | Second dose  N=5158 |
| --- | --- | --- |
| Patients reported AEs | 677（12.5%） | 464（9.0%） |
| Local reactions |  |  |
| Local pain  Any  Grade3/4 | 325（6.0%）  2（＜0.1%） | 175（3.4%）  2（＜0.1%） |
| Local swelling  Any  Grade3/4 | 31（0.6%）  2（＜0.1%） | 32（0.6%）  2（＜0.1%） |
| Subcutaneous nodules  Any  Grade3/4 | 27（0.5%）  2（＜0.1%） | 14（0.3%）  2（＜0.1%） |
| Systemic reaction |  |  |
| Fever  Any  ≥38℃ | 44（0.8%）  15（0.3%） | 12（0.2%）  4（0.1%） |
| Headache  Any  Grade3/4 | 53（1.0%）  1（＜0.1%） | 36（0.7%）  2（＜0.1%） |
| Fatigue  Any  Grade3/4 | 178（3.3%）  1（＜0.1%） | 129（2.5%）  2（＜0.1%） |
| Muscle soreness  Any  Grade3/4 | 77（1.4%）  2（＜0.1%） | 70（1.4%）  1（＜0.1%） |
| Joint pain  Any  Grade3/4 | 24（0.4%）  3（0.1%） | 16（0.3%）  2（＜0.1%） |
| Nausea  Any  Grade3/4 | 35（0.6%）  2（＜0.1%） | 15（0.3%）  1（＜0.1%） |
| Loss of appetite  Any  Grade3/4 | 17（0.3%）  1（＜0.1%） | 6（0.1%）  2（＜0.1%） |
| Anaphylaxis  Any  Grade3/4 | 9（0.2%）  0 | 8（0.2%）  0 |
| Dizzy  Any  Grade3/4 | 9（0.2%）  0 | 6（0.1%）  0 |
| Disturbance in respiration  Any  Grade3/4 | 4（0.1%）  0 | 2（＜0.1%）  0 |
| Breast pain  Any  Grade3/4 | 3（0.1%）  0 | 5（0.1%）  0 |
| Thirsty  Any  Grade3/4 | 4（0.1%）  0 | 2（＜0.1%）  0 |
| Diarrhea  Any  Grade3/4 | 2（＜0.1%）  1（＜0.1%） | 2（＜0.1%）  1 |
| Angina pectoris  Any  Grade3/4 | 4（0.1%）  1（＜0.1%） | 1（＜0.1%）  0 |
| Others  Any  Grade3/4 | 21（0.4%）  0 | 23（0.4%）  0 |

Table S2. multivariate analysis of AEs in vaccinated population.

| Clinical statistics | Factors | OR，95%CI |
| --- | --- | --- |
| Age | ≤45（ref）  ＞45 | 0.914（0.777-1.075） |
| Medium-high risk resisdence history | Yes（ref）  No | 0.671（0.173-2.598） |
| Chronic illness | NO（ref）  Have | 0.895（0.737-1.087） |
| Level of education | With bachelor degree (ref)  Without bachelor degree | 0.794（0.680-0.927） |
| T stage | T1-2  T3-4 | 0.903(0.655-1.246) |
| N stage | N0（ref）  N1-3 | 1.081（0.912-1.283） |
| M stage | M0（ref）  M1 | 0.724（0.398-1.317） |
| HR status | Negative（ref）  Positive | 0.977（0.812-1.177） |
| HER2 status | Negative（ref）  Positive | 1.138（0.958-1.352） |
| Previous neoadjuvant | NO（ref）  Yes | 0.931(0.758-1.144) |
| Previous surgery | NO（ref）  Yes | 0.995（0.732-1.370） |
| Previous adjuvant | NO（ref）  Yes | 0.925（0.730-1.172） |
| Metastases | NO（ref）  Yes | 1.310（0.967-1.773） |
| Type of vaccines | Inactivated Virus (ref)  Other viruses | 0.937（0.689-1.273） |
| Time window of vaccination | Vaccinated after diagnosis (ref)  Vaccinated before diagnosis | 0.919（0.785-1.077） |
